# Charging of DNA Complexes in Positive-Mode Native Electrospray Ionization Mass Spectrometry

**DOI:** 10.1021/jasms.4c00335

**Published:** 2024-10-17

**Authors:** Mia L. Abramsson, Louise J. Persson, Frank Sobott, Erik G. Marklund, Michael Landreh

**Affiliations:** † Department of Microbiology, Tumor and Cell Biology, Karolinska Institutet, 171 65 Solna, Sweden; ‡ Department of Chemistry - BMC, Uppsala University, 751 23, Uppsala, Sweden; § Astbury Centre for Structural Molecular Biology, School of Molecular and Cellular Biology, Faculty of Biological Sciences, University of Leeds, LS2 9JT Leeds, U.K.; ∥ Department for Cell and Molecular Biology, Uppsala University, 751 24 Uppsala, Sweden

**Keywords:** protein−DNA complex, charge state distribution, electrospray ionization

## Abstract

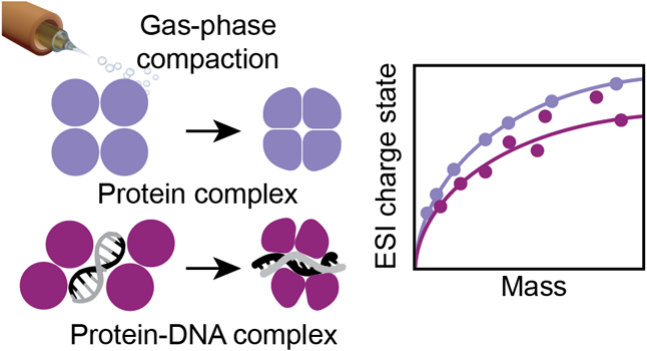

Native mass spectrometry (nMS) provides insights into the structures and dynamics of biomacromolecules in their native-like states by preserving noncovalent interactions through “soft” electrospray ionization (ESI). For native proteins, the number of charges that are acquired scales with the surface area and mass. Here, we explore the effect of highly negatively charged DNA on the ESI charge of protein complexes and find a reduction of the mass-to-charge ratio as well as a greater variation. The charge state distributions of pure DNA assemblies show a lower mass-to-charge ratio than proteins due to their greater density in the gas phase, whereas the charge of protein–DNA complexes can additionally be influenced by the distribution of the ESI charges, ion pairing events, and collapse of the DNA components. Our findings suggest that structural features of protein–DNA complexes can result in lower charge states than expected for proteins.

## Introduction

Native mass spectrometry (nMS) allows the study of biomolecules in their native-like states. Using positive electrospray ionization (ESI) to preserve the noncovalent interactions and the overall conformation of proteins, protein complexes, and nucleic acids allows us to investigate their structures, dynamics, and interactions in vacuum. The charge state distribution (CSD) of a protein complex can provide insights into its conformation, as the number of charges differs between compactly folded and disordered proteins.^1,2^ This difference implies that conformational changes can be monitored through CSD shifts.^3−5^

Folded proteins display a strong empirical correlation between the surface area and the number of ESI charges.^6,7^ Since most compact protein ions are roughly spherical, their average ESI charge can be estimated based on their mass.^6,8^ Importantly, the number of charges in positive or negative ionization mode is not affected by the protein’s solution charge, as artificial proteins with no ionizable residues, as well as proteins with a large excess of either basic or acidic residues, display near-identical CSDs.^8,9^ On the other hand, the CSDs of these proteins show different sensitivities to solution additives or gas-phase collisions, indicating a role for proton affinity despite the obvious robustness of the charging mechanism.^10,11^ These findings lead us to ask whether it is also insensitive to the presence of highly charged nonprotein components in a molecular complex. Specifically, we turned to complexes containing DNA molecules, which carry a high negative solution charge due to the phosphate groups in the backbone. Proteins bind to DNA via strong electrostatic interactions, making them particularly suitable for nMS analysis. In fact, protein–DNA complexes are among the earliest examples of noncovalent complexes that could be observed in the gas phase.^12−15^ Being able to predict the CSDs of protein–DNA complexes may therefore offer insights into their conformational landscape. However, while charge predictions based on mass are relatively straightforward for protein complexes due to their relatively constant gas phase density, other molecules can differ significantly. Ion mobility measurements of small molecules are pronounced, McLean and co-workers showed for singly charged ions that the correlation between collision cross-section and mass differs significantly between lipids, peptides, carbohydrates, and nucleotides.^16,17^ Strikingly, nucleotides showed the highest density, i.e., the smallest collision-cross-section relative to their mass. These findings strongly suggest that the presence of a more dense component in a multicomponent complex will impact its ESI charge state.

## Experimental Section and Results

**Figure 1:**
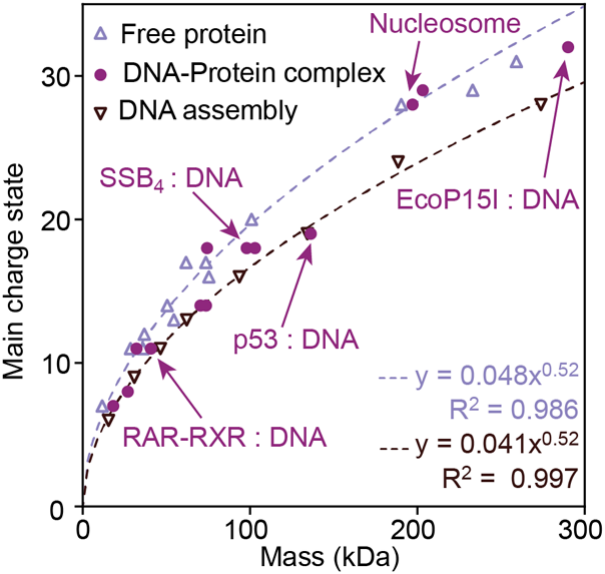
Different CSDs of protein and DNA complexes can be explained by their different gas-phase densities. The correlations between mass and main charge state for protein-only and DNA-only complexes were fitted as described in the text. Mixed protein–DNA complexes taken from the same studies as the protein complexes display a greater variation in the number of charges than those of compact protein or DNA complexes. The five protein–DNA complexes shown in Figure 2 are indicated by arrows. The *R*^2^ value is adjusted for the number of data points and parameters.

As the first step, we surveyed the literature for recent examples of protein and DNA complexes recorded in positive mode and using ammonium acetate solutions as a solvent. From these reports, we summarized the masses of the DNA and protein components in each complex. Determining the average charge would require access to the raw data, so to estimate the charge, we used the main charge state, the most intense charge state at, or close to, the center of the charge state envelope (Table 1). Comparisons between CSDs of DNA and proteins are limited because DNA molecules are commonly analyzed in negative ionization mode.^18,19^ However, Sobott and co-workers recently used nMS in positive ionization mode at varying concentrations of ammonium acetate to analyze nanopores assembled from DNA molecules and which fall into the same mass range as most protein complexes (Table S1).^20^ Using the experimental CSDs for DNA assemblies, protein–DNA complexes, and the corresponding free proteins from 15 to 300 kDa, we plotted the most abundant charge states of each species as a function of their masses (Figure 1). Free proteins follow the established power law relationship between mass (*m*) and charge (*z*), which we determined for the main charge state to be eq 1.
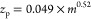
1where the subscript p is for “protein”. The curve is slightly flatter than in previous reports;^6^ however, these earlier data sets included only proteins below a mass of 50 kDa. We then plotted the masses and charges of the DNA nanopores (Figure S1). We observe an exponential mass–charge relationship, shown in eq 2.
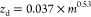
2Where the subscript d is for “DNA”. We note that the exponents in eqs 1 and 2 are very similar, so we made an attempt to fit both DNA and protein at the same time and allow the prefactors to be different but forcing the exponents to be the same (see Supporting Information for parameter fitting details). This resulted in the following expressions:
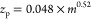
3
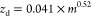
4

**Table 1:** Extracted Mass Based on Protein and Nucleotide Sequence or Reported Theoretical Mass and Most Intense Charge State for Protein–DNA Complexes and the Corresponding Free Proteins Used in This Study

		mass (Da)				main charge state		
protein name	oligomeric state	protein	DNA	DNA Complex	% DNA	protein only	DNA complex	reference
BirA	monomer	36,771	29,331	66,102	44	12	15	(26)
	dimer	73,559	29,331	102,890	28	17	18	
EthR	dimer	50,475	23,153	73,628	31	14	14	(27)
	tetramer	100,852	38,770	139,622	28	20	22	
	hexamer	151,278	38,770	190,048	20	n.d.	24	
nucleosome	octamer	106,220	90,873	197,093	46	n.d.	28	(28)
gp32	monomer	28,500	3588	32,088	11	11	11	(29)
HMGA2	monomer	11,600	15,300	26,900	57	7	8	(30)
	monomer	11,600	6600	18,200	36	n.d.	7	
FraR	dimer	54,408	15,932	70,340	22	13	14	(31)
thrombin	monomer	36,006	4723	40,730	11	11	11	(32)
RAR-RXR	heterodimer	20,843	13,111	33,954	38	n.d.	13	(33)
EcoP15I	heterotrimer	259,145	30,910	290,055	10	31	32	(34)
p53	tetramer	120,300	15,946	136,246	12	n.d.	19	(35)
MutS	dimer	190,322	12,869	203,191	6	28	29	(36)
SSB_4_	homotetramer	75,372	22,589	97,961	23	16	18	(37)
p50 (NF-κB)	dimer	61,715	12,560	74,275	17	17	19	(38)

Equations 3 and 4 fit the data well, with a similar or higher adjusted *R*^2^ than eqs 1 and 2 (Table S2). The relative uncertainty is quite high for the prefactors in eqs 2–4, but the exponent is well-defined by the data to within a few percent (Table S2).

Next, we considered the origin of the ESI charge. Biomolecules are released from an electrospray droplet that can carry a net charge up to the Rayleigh limit. The limit is given by the surface area of the droplet, which, in turn, is uniquely determined by the radius or volume under the assumption that the droplet is spherical. In the later stages of the ESI process, the solvent evaporates until the droplet barely encapsulates the analyte; hence, its final size and charge-carrying capacity are determined by the physical extent of the analyte. One can formulate the expected relationship between charge and volume (*V*).
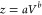
5

The coefficients *a* and *b* should both be universal, at least for molecules that do not vary widely in their shapes. From the Rayleigh stability limit and because the mass of an object is proportional to the volume, one would conclude that *b* should be 1/2. Similarly, *a* should have a well-defined value rooted in the fundamental constants and solvent properties. However, empirical evidence from both the literature^6^ and eqs 3 and 4 points toward a slightly higher value for *b*, which influences the value of the prefactor *a*. The reason behind this might be that the surface area is more important than the radius or the volume at the final stages of CRM, and that the former becomes more feature-rich with increasing molecular size. It may also be that larger macromolecules allow for more cavities that increase in size beyond the volume actually occupied by their atoms. Regardless of the cause, we acknowledge that *b* might deviate from 1/2 and thus treat both *a* and *b* as free parameters and determine their values by fitting our model to the data. We see in Figures 1 and S1 that both the DNA and protein data fit well with the same value for *b*, even if other types of analytes might, in principle, require other values. With MS, we cannot measure the molecular volume confidently. Ion mobility MS is probably the best option, but the volumes can only be indirectly inferred and they are inconsistent with established densities for proteins due to the assumptions made.^21^ In contrast, we can infer the mass with high precision from MS, and for a specific class of analytes, such as proteins, it gives us the volume because they all have approximately the same density: *V* = *m*/ρ. This allows us to rewrite eq 4 as the familiar relation between *z* and *m* (as seen in eqs 1 and 2):
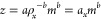
6where *a*_*x*_ = *aρ*_*x*_^–*b*^. The constant *a*_*x*_ is specific for an analyte class *x*, because it contains the density factor. We can now compare the charge picked up by proteins and DNA in ESI:
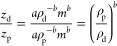
7where the subscripts p and d denote protein and DNA. Interestingly, the quotient is entirely dependent on the densities and *b*. Using the densities ρ_p_ and ρ_d_, we can use eq 7 to predict how different the expected *z* is between DNA and proteins. Different sources claim slightly different density values, but using ρ_p_ = 1.35 g/cm^3^, which is consistent with the studies referenced by Fischer and co-workers,^22^ and ρ_d_ = 1.7 g/cm^3^ from Schildkraut and co-workers,^23^ together with *b* = 0.52 (eqs 3 and 4) we get *z*_d_/*z*_p_ = 0.89, meaning that the DNA complexes pick up almost 11% less charge than proteins of the same mass as a direct consequence of their different densities. One can rewrite the first part of eq 7 as
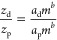
8

Importantly, while eq 7 yields the charge difference using the densities as input together with the exponent *b*, eq 8 takes no such input, and the charge difference it yields stems straight from the experimental data. Using eq 8 with the *a*_*x*_ values from eqs 3 and 4 to get *z*_d_/*z*_p_, we find that the DNA charges are 15% below those of proteins with the same mass. While the prefactors themselves come with considerable uncertainty, they are strongly correlated, so their quotient is consequently well-defined with a 95% confidence interval of 12% to 18% (Table S3). The close agreement between the results from eqs 7 and 8 corroborates our idea that the lower charging of DNA can be largely explained by the density difference between protein and DNA. With the caveat that the DNA data stem from a single study, we conclude that we can with the simple density adjustment predict the ESI charge states of DNA complexes in positive mode. Our observations confirm the first reports by Loo and co-workers that DNA molecules are subject to the same ion pairing and charging mechanisms as proteins, which completely mitigates the difference in solution charge.^24,25^

Next, we applied these considerations to mixed complexes that contain both protein and DNA. Interestingly, we find variations in the correlation between the mass and charge. As evident from Figure 1, several of the protein–DNA complexes acquire a lower charge than expected for a protein-only complex of the same mass. For some examples, such as the DNA-bound p53 tetramer, the mass-to-charge ratio approaches that of the DNA oligomers. On the other hand, large complexes with DNA (>150 kDa), for example nucleosomes, appeared to charge the same as protein-only complexes (Figure 1). We calculated the relative DNA content of each complex as a fraction of the total mass (Table 1). The two largest DNA-containing complexes, EcoP15I and the *Xenopus* nucleosome, contain 10% and 46% DNA, respectively, but charge essentially the same as free proteins (Figure 1), strongly indicating that the higher density of the DNA components is not the sole reason for variations in the ESI charge.

## Discussion

**Figure 2:**
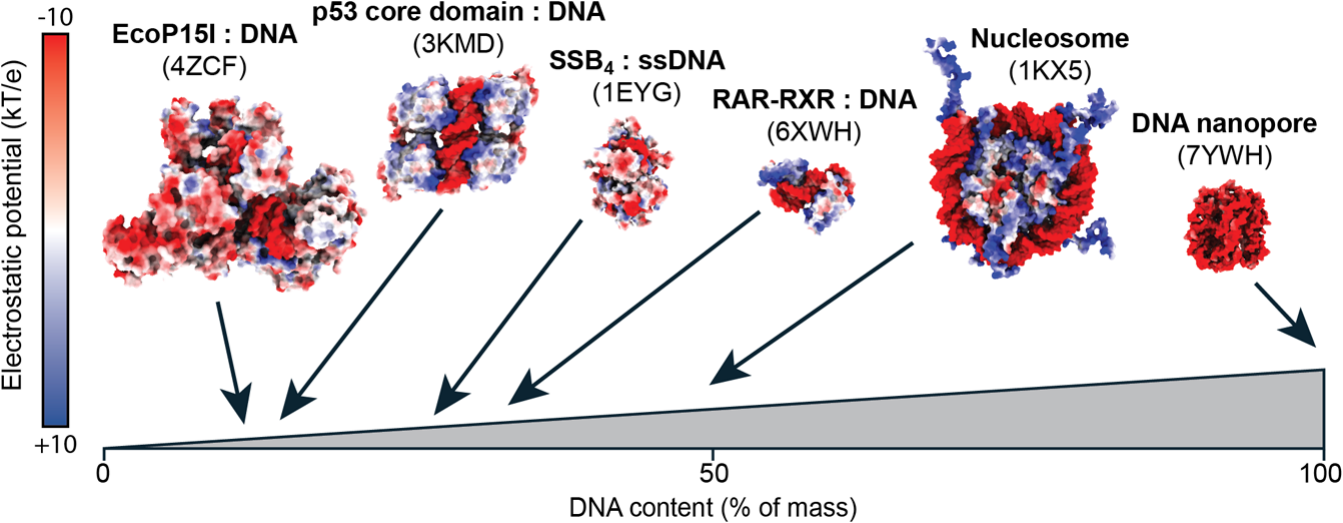
Relative DNA content and surface electrostatic potential (red negative potential, blue positive potential) renderings of the high-resolution structures of DNA complexes analyzed by nMS.

So what may be the reason some of the protein–DNA complexes exhibit slightly lower charges? Gabelica and co-workers used ion mobility MS to compare the solution and gas-phase structures of DNA duplexes ionized in negative mode.^39^ MD simulations revealed that the charge neutralization of the phosphate backbone during ESI reduces Coulombic repulsion and allows for structural rearrangements. The resulting compaction of >20% relative to the solution structures is significantly greater than the average compaction reported for proteins, which is usually below 10%.^40^ This effect may be exacerbated by the spatial distribution of the ESI charges. In a protein, these charges are distributed evenly over multiple sites on the surface, usually basic residues, which can be neutral or positively charged. However, an uneven distribution of the charges affects the conformational stability of the protein in the gas phase.^9^ In DNA, we can assume a more uneven distribution than that in proteins. Some protein–DNA complexes exhibit relatively large DNA surfaces (Figure 2). Once neutralized by ion pairing mechanisms during ionization in positive mode, the DNA backbone likely has a lower proton affinity than basic side chains on the neighboring protein surfaces.^41^ While the total number of ESI charges scales with the mass of the complex, these charges could preferentially attach to the protein surfaces, which would lead to an uneven distribution of the charges in the complex. In addition, charge neutralization during ESI can involve pairing between positively and negatively charged groups.^42^ In protein–DNA complexes, basic residues could contribute to neutralization of the DNA backbone, which would promote compaction of the complex through additional intermolecular contacts.

On the basis of our findings, we believe that the propensity of DNA molecules to collapse during charge neutralization, an uneven distribution of the ESI charges, and structural rearrangements due to ion pairing between DNA and protein all promote partial collapse during the last stages of ionization. While the contributions from each of these events are difficult to estimate, they are all related to the fact that the negative solution charge of the DNA is neutralized during positive-mode ESI and significantly reduced during negative-mode ESI. The extent to which they distort the mass-to-charge ratio may depend on the architecture of the complex. In the tetrameric DNA binding domain of p53, the four folded subunits are arranged around a central double helix (Figure 2), whose collapse may lead to additional compaction of the whole complex. Similarly, the subunits in the RAR–RXR complex, which also charges less than expected, are held apart by DNA. The collapse of such an “inner DNA skeleton” could give rise to a more compact conformation and, consequently a lower charge. In the nucleosome, on the other hand, the double helix is located on the outside (Figure 2), which when compacted could lead to a strongly charge-stabilized assembly. According to the above scenarios, the location of the DNA moiety could determine whether a complex charges closer to a protein or a DNA assembly, offering a potential structural insight.

## Conclusions

These observations extend our previous findings that the charge of a protein complex in nMS is essentially unrelated to its surface properties, demonstrating that the same mass-to-charge correlation holds for complexes composed of protein or DNA. The robustness of the ESI charging process means that one can readily predict the average charge states for DNA complexes. We find that by taking their higher density into account, the charges of DNA assemblies can be predicted in the same way as for proteins. Protein–DNA complexes, however, display a greater variation in their mass-to-charge ratios due to ion pairing, structural collapse, and uneven distribution of ESI charges on their surface. In summary, we show that the ESI process can have a stronger effect on the gas phase structure of protein–DNA complexes than on proteins, as it involves a pronounced deviation from the native solution charge.

## Supporting Information Available

Supplementary tables, equations for the fitting parameters, and additional references (PDF)
