# Supplementary material for: Charging of DNA Complexes in Positive-Mode Native Electrospray Ionization Mass Spectrometry
Source: J Am Soc Mass Spectrom. 2024 Oct 17;35(12):3157–62. doi: 10.1021/jasms.4c00335 (PMC11622369; doi:10.1021/jasms.4c00335)
Supplement: Supplementary file 1 — js4c00335_si_001.pdf [file js4c00335_si_001.pdf]

Supplementary information for

# Charging of DNA Complexes in Positive-Mode Native Electrospray Ionization Mass Spectrometry

Mia L. Abramsson, Louise J. Persson, Frank Sobott, Erik G. Marklund*, and Michael Landreh*

1 Department of Microbiology, Tumor and Cell Biology, Karolinska Institutet, 171 65 Solna, Sweden

2 Department of Chemistry - BMC, Uppsala University, 751 23, Uppsala, Sweden

3 Astbury Centre for Structural Molecular Biology, School of Molecular and Cellular Biology, Faculty of Biological Sciences, University of Leeds, LS2 9JT Leeds, U.K.

4 Department for Cell and Molecular Biology, Uppsala University, 751 24 Uppsala, Sweden

* Correspondence to michael.landreh@icm.uu.se or erik.marklund@kemi.uu.se

**Document contains:**

Supplementary Tables S1, S2

Supplementary Figure S1

Parameter fitting

Supplementary References

***Table S1.*** *DNA oligomeric states, measured mass, and main charge states for DNA oligomers reported by Sobott et al.*[1]

| Oligomeric state | Mass (Da) | Main charge state |
|---|---|---|
| 1 | 15,392.4 | 6 |
| 2 | 30,763.8 | 9 |
| 3 | 46,396.9 | 11 |
| 4 | 62,069.8 | 13 |
| 6 | 93,560 | 16 |
| 9 | 134,225.5 | 19 |
| 12 | 188,457.6 | 24 |
| 18 | 273,781.2 | 28 |

***Table S2.*** *Parameters and adjusted $R^2$ after fitting Eqs. S1 and S2 to the MS-derived data for the pure proteins and pure DNA in Tables 1 and S1. 95% confidence intervals are given in parentheses.*

| | **Protein only (Eq. S1)** | **DNA only (Eq. S1)** | **Protein and DNA (Eq. S2)** |
|---|---|---|---|
| $a_p$ | 0.049<br>(0.031–0.076) | - | 0.048<br>(0.032-0.066) |
| $a_d$ | - | 0.037<br>(0.028-0.046) | 0.041<br>(0.028-0.057) |
| $b$ | 0.52<br>(0.48-0.56) | 0.53<br>(0.51-0.56) | 0.52<br>(0.49-0.55) |
| $a_d/a_p$ | - | - | 0.85<br>(0.823–0.88) |
| $R^2$ | 0.986 | 0.997 | 0.990 |
| ${R^2}_p$ | - | - | 0.986 |
| ${R^2}_d$ | - | - | 0.997 |

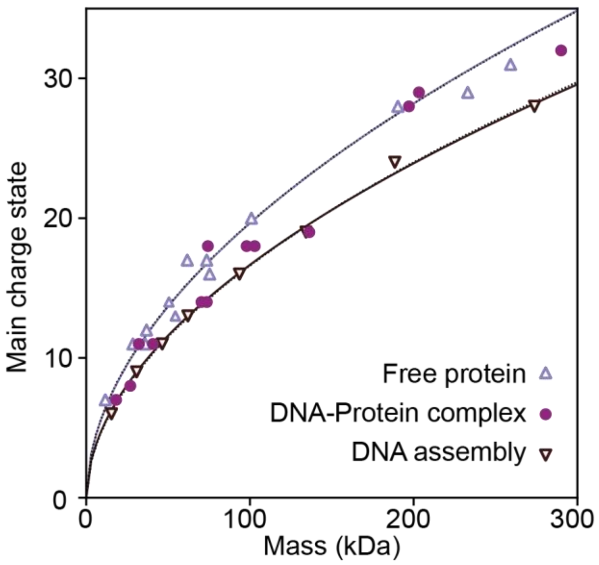


**Figure S1.** Fitting the model separately to protein and DNA data (dotted lines), and to both at the same time with a common exponent *b* (solid lines). Both approaches yield virtually indistinguishable lines, which gives strong support for using the same exponent for both protein and DNA, validating the assumption of a universal *b* in our model.

## Parameter fitting

In order to fit the function

$$z = am^b \quad \textbf{(S1)}$$

to the mass and charge data, we used unconstrained least-squares optimization as implemented in the function curve_fit from the SciPy python package[2]. Fitting the function separately to the protein and DNA data is trivial, and could be done without special considerations. We also fitted the model both the DNA and protein data simultaneously (for clarity, not the protein-DNA complexes but the pure protein and pure DNA data) and allowed separate prefactors $a$ for protein and DNA, but forced the exponent $b$ to be the same for both, which required modifications to the model to enable the fitting. Specifically, we instead fitted the following expression:

$$z = ka_p m^b + (1-k)a_d m^b \quad \textbf{(S2)}$$

where $k = 1$ for proteins and $k = 0$ for DNA. As such, proteins will only have an impact on the first term and DNA on the other. Because $b$ appears in both terms, it on the other hand will be optimized using data from both protein and DNA.

To assess the goodness of fit we calculated the adjusted $R^2$ for the three fits (protein only, DNA only, both), because the protein-only and DNA-only fits (Eq. S1) use two parameters while the combined fit (Eq. S2) uses three. Additionally, we calculated the adjusted $R^2$ using the protein and the DNA data points separately, using the parameters obtained from the combined fit. The latter adjusted $R^2$ values were denoted $R^2_p$ and $R^2_d$. For each parameter of the three fits, we also computed 95% confidence intervals using the bootstrap function of SciPy[2]. The bootstrap procedure applied the bias-corrected and accelerated method (BCa), with 10,000 resamples of batches matching the size of the full data set, sampled with replacement.

The fitted parameters, confidence intervals and adjusted $R^2$ are provided in Table S2. Reassuringly, the exponent $b$ was very similar in the separate fits to the protein data and the DNA data, displaying narrow and similar confidence intervals, and yielded virtually identical lines when plotted (Fig. S1). This motivated using Eq. S2 as it is a more general model with fewer parameters in total compared with using Eq. S1

separately on the protein and DNA data to optimize a total of four parameters. Eq. S2 being a good choice is reaffirmed by the adjusted $R^2$ of its fit to the data being at the same high levels as the fit to Eq. S1.

The confidence intervals for the prefactors $a_p$ and $a_d$ were broader than for the exponent $b$, likely due to the relatively greater impact the exponent has on the fit. The ratio $a_p/a_d$ on the other hand had a narrow confidence interval, despide $a_p$ and $a_d$ having broad ones. The reason is likely that they are both anti-correlated with $b$: a fit that yields a large $b$ will result in low $a_p$ and $a_d$, so that both the numerator and the denominator fluctuate in concert. As such, we can use the quotient to estimate the difference in the charge of proteins and DNA with high certainty, as described in the main text. However, the large uncertainties for the prefactors means that the universal constant $a$ is difficult to determine confidently, even with a highly accurate value for the density of proteins or DNA.

## References


(1) van Dyck, J. F.; Burns, J. R.; Le Huray, K. I. P.; Konijnenberg, A.; Howorka, S.; Sobott, F. Sizing up DNA Nanostructure Assembly with Native Mass Spectrometry and Ion Mobility. *Nat. Commun.* **2022**, *13* (1), 3610. https://doi.org/10.1038/s41467-022-31029-5.

(2) Virtanen, P.; Gommers, R.; Oliphant, T. E.; Haberland, M.; Reddy, T.; Cournapeau, D.; Burovski, E.; Peterson, P.; Weckesser, W.; Bright, J.; van der Walt, S. J.; Brett, M.; Wilson, J.; Millman, K. J.; Mayorov, N.; Nelson, A. R. J.; Jones, E.; Kern, R.; Larson, E.; Carey, C. J.; Polat, İ.; Feng, Y.; Moore, E. W.; VanderPlas, J.; Laxalde, D.; Perktold, J.; Cimrman, R.; Henriksen, I.; Quintero, E. A.; Harris, C. R.; Archibald, A. M.; Ribeiro, A. H.; Pedregosa, F.; van Mulbregt, P. SciPy 1.0: Fundamental Algorithms for Scientific Computing in Python. *Nat. Methods* **2020**, *17* (3), 261–272. https://doi.org/10.1038/s41592-019-0686-2.
